# Dynamical links of convective storms associated with tropospheric biennial oscillation in the Indian monsoon regime

**DOI:** 10.1038/s41598-022-15772-9

**Published:** 2022-07-14

**Authors:** U. V. Murali Krishna, Subrata Kumar Das, K. N. Uma, Abhishek Kumar Jha, G. Pandithurai

**Affiliations:** 1 Indian Institute of Tropical Meteorology, Ministry of Earth Sciences, Pune, 411008 India; 2 Space Physics Laboratory, Vikram Sarabhai Space Centre, Trivandrum, 695022 India; 3 Department of Atmospheric and Space Sciences, Savitribai Phule Pune University, Pune, 411007 India

**Keywords:** Atmospheric science, Atmospheric dynamics

## Abstract

Tropospheric Biennial Oscillation (TBO) is characterized by a tendency for a relatively stronger monsoon to be followed by a relatively weaker one (positive) or vice-versa (negative). This study examines the distribution of different convective systems occurring during TBO phases over the Indian monsoon region. During negative TBO phase, convection is preferential over the Arabian Sea (AS), whereas during positive TBO phase, it is favoured over the land areas and Bay of Bengal (BoB). The isolated shallow convection (ISC) is dominated over the AS and Indian west coast during negative TBO years. A relatively stable environment (statically) capped with drier mid-troposphere results in abundant ISC over the AS. Broad stratiform rain (BSR) dominates over the central and east coast of India, BoB and Myanmar coast during positive TBO years and wide convective core (WCC) are present along the orographic regions, i.e., Myanmar coast and Western Ghats during negative TBO phase. The anomalous easterlies induced by the upper-ocean temperature gradient interact with the mean monsoon winds during positive TBO to provide pathways for developing BSR echoes. The deep-wide convection (DWC) are higher along the Himalayan foothills during positive TBO years. The moist low-level flow from the AS is trapped by dry mid-level flow from high latitudes, resulting in orographic lifting along the Himalayan foothills and form DWC.

## Introduction

Mesoscale convective systems (MCSs) are significant rain-producing weather phenomena, especially in the tropics. MCS brings more than 50$\%$ of rainfall in the tropics^1^. MCSs are characterized by both stratiform and convective regions of precipitation. They provide an important link between convection and large scale atmospheric circulation. Different precipitating systems are associated with different heating profiles and mass transport^2^, significantly impacting global circulation^3^. Apart from the MCSs, warm rain processes also contribute significantly to the Indian summer monsoon (ISM, June to September)^4–7^. Detailed analysis on the spatial and temporal variability of convective systems reveals their evolution and lifetime, which helps to improve rainfall retrievals and climate predictions. The knowledge of the spatial structure of convection has been revolutionized with the availability of Tropical Rainfall Measuring Mission (TRMM) Precipitation Radar (PR) measurements^8–15^.

The ISM exhibits large spatial variability both in convection and rainfall. There have been studies on the spatial variability of different convective systems over Indian region and surrounding oceans in the recent past. For example, intense convection occurs near the mountain regions^9^. MCSs develop significant stratiform rain in the eastern parts of the Indian subcontinent^10^. In addition to MCSs, cumulus/congestus clouds are favourable over the western parts of the Indian subcontinent^7^. Thus, the ISM exhibits wide spectrum of convection, from shallow to deep convection, and stratiform rain^16^. Houze et al.^10^ analyzed the three-dimensional structure of different convective systems over the Himalayan region, limiting two monsoon seasons (2002–2003). Romatschke et al.^11^ examined extreme convection’s temporal and spatial variability over the south Asian region using 8 years of TRMM-PR data. They revealed that deep convection prevailed on the east coast of India during the pre-monsoon season, whereas it is dominated in the western foothills of Himalayas during the monsoon. Romatschke and Houze^12^ studied convective and stratiform precipitation characteristics over different areas of the ISM. They found that convective systems producing rainfall differ in the coastal, oceanic and orographic regions. Bhat and Kumar^17^ studied the vertical structure of intense convective clouds over the south Asian region and reported that the cumulonimbus towers occur more frequently over the foothills of Himalayas. Tawde and Singh^18^ analyzed the spatial patterns of monsoon rainfall with the orography of Western Ghats (WG). They found that the orographic precipitation depends on the topography and steepness of the windward slopes of the mountain. Shige and Kummerow^19^ investigated the precipitation top heights associated with the orographic rainfall in the Asian monsoon region. They observed shallow precipitating top heights over the WG. Recently, Krishna et al.^20^ studied the diurnal cycle of convective storms over WG and revealed that the shallow top height over coastal and oceanic regions, where as deep and intense convection prevails over high altitudes. Using disdrometer measurements, Murali Krishna et al.^21^ hypothesized the dominance of shallow convection over the ocean and windward sides, while deeper storms on the leeward side of the WG mountains. These studies emphasized the spatial variability of convective systems, and hence the underlying mechanisms are different over the Indian subcontinent.

The ISM rainfall also shows large temporal variability ranging from intraseasonal to interannual time scales. Moreover, the ISM also involves coupled land-atmosphere-ocean interactions and has strong links with global oscillations like Tropospheric Biennial Oscillation (TBO)^22,23^ and El Niño Southern Oscillations (ENSO)^24^. TBO is an integral part of the coupled ocean-atmosphere system^22,25^, which significantly impacts the strength of the Asian monsoon. TBO is defined as the tendency for a relatively strong monsoon followed by a relatively weak one and vice versa^22^. The transition occurs in spring for the south Asian or Indian monsoon. TBO is not so much an oscillation, but it tends to flip-flop back and forth from year to year. TBO involves large-scale atmospheric east-west circulations in the tropics, convective heating anomalies over Africa and the Pacific, and tropical mid-latitude interactions in the Northern Hemisphere. Loschnigg et al.^26^ suggested that the anomalous sea surface temperature (SST) over the tropical Indian Ocean is responsible for the TBO. Pillai and Mohankumar^27^ demonstrated that the Indian Ocean Dipole (IOD) is an important local forcing for the TBO. The spatial structures and the mechanism for the transition of TBO phases are well documented^22,23,26^.

Several researchers detected the TBO signals in the rainfall over Asia^28,29^, especially rainfall over the Indian subcontinent^30,31^. TBO signals are also seen in the tropical circulation, SST, and upper-ocean heat content^31,32^. Li et al.^23^ examined the structure and seasonal evolution characteristics of the TBO using the National Centre for Environmental Prediction (NCEP)/National Centre for Atmospheric Research (NCAR) reanalysis products. They noted that the significant convective regions associated with the TBO are the southeast Indian Ocean and the western North Pacific. Several researchers^22,23,33^ studied the possible linkages between TBO and ENSO. Meehl et al.^33^ observed the weakening of TBO signal without any influence of ENSO in the Asian-Australian monsoon region. Li et al.^34^ demonstrated the effect of TBO on the Indian Ocean without any remote forcing such as ENSO.

Numerous attempts were made to simulate the TBO by considering the ocean-atmosphere feedback within the tropics. Rajeevan and Nanjundiah^35^ reviewed TBO rainfall over south Asia using the Coupled Model Inter-comparison Project (CMIP3). Li et al.^36^ simulated the transition from strong/weak ISM to strong/weak Australian monsoon associated with the TBO using CMIP3 and CMIP5 models. Their study demonstrated that the Indian-Australian monsoon link is robust at the TBO timescale. The spatial patterns of the summer monsoon rainfall associated with the TBO are simulated using Atmospheric Model Inter-comparison Project (AMIP)^37^. They suggested that the SST anomalies can enhance the rainfall variability in the East Asian summer monsoon. Konda et al.^38^ examined the spatial pattern of TBO rainfall using the NCEP climate forecast system (CFSv2) hindcast. Their results showed that the CFSv2 model could capture the observed rainfall anomalies associated with positive TBO years over the south Asian region. However, the model is unable to capture the rainfall anomalies associated with negative TBO years. Unless there is a clear understanding about different types of convection predominantly occurring during different TBO phases, the model simulations may not able to represent the observed spatial structures of rainfall associated with the TBO. This motivated us to understand how the convective systems evolved in space with different TBO phases over the Indian subcontinent.

The spatial rainfall patterns are different during contrasting TBO phases related to the strength of the Walker circulation, anomalous Asian land surface temperatures, Pacific and Indian Ocean SST anomalies. These large-scale features modify the local dynamical and thermodynamical phenomena that affect the convective activity’s strength. However, the spatial patterns of different convective systems and the underlying physical mechanisms are not examined, specifically over India and adjacent ocean regions. The present study examines this gap area using the TRMM precipitation radar measurements and reanalysis data over the Indian monsoon region.

## Results and discussion

### TRMM climatology of convective systems over Indian subcontinent

**Figure 1:**
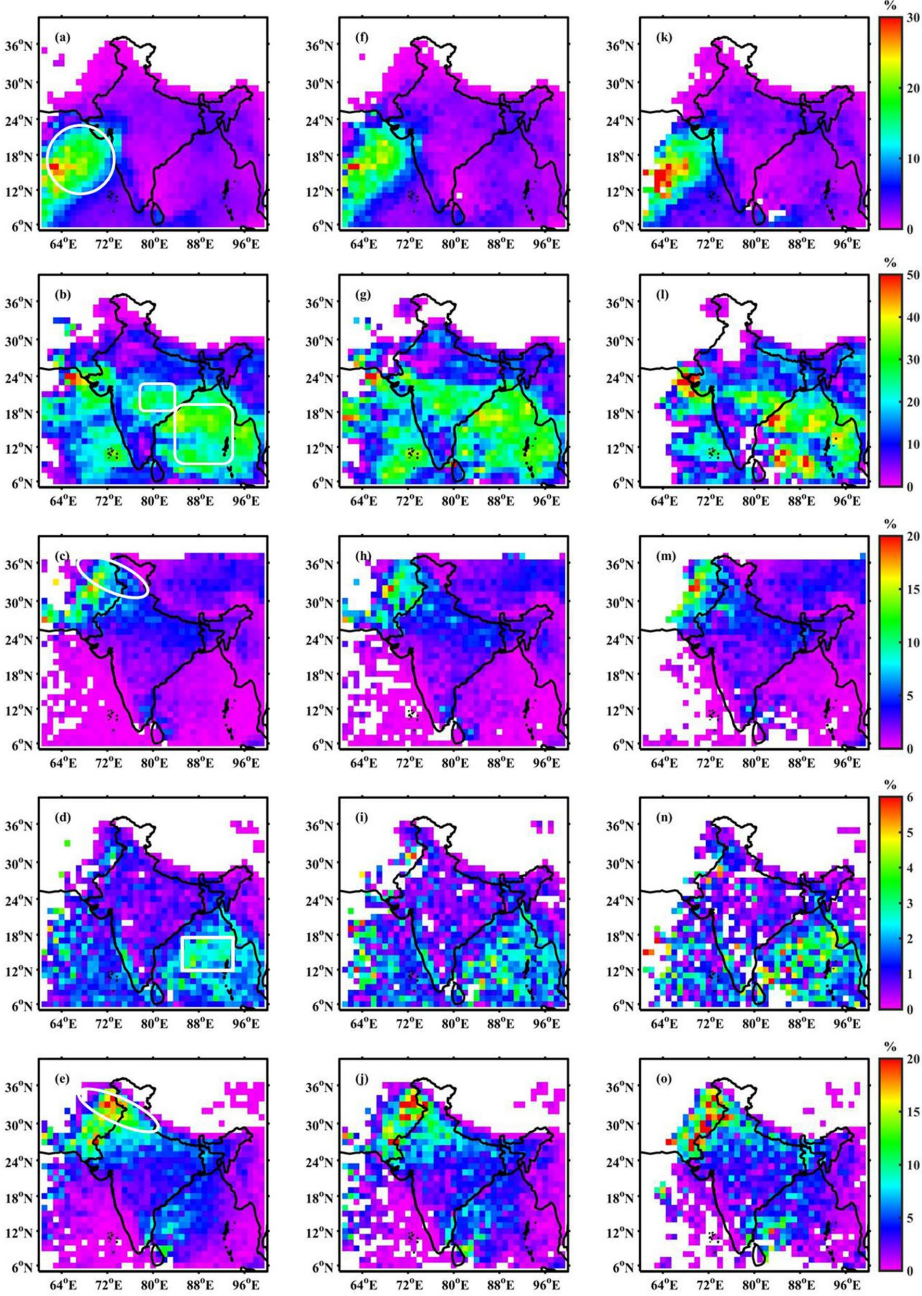
The climatological (1998–2013) occurrence of (**a**) ISC, (**b**) BSR, (**c**) DCC, (**d**) WCC, and (**e**) DWC echoes. The major regions of occurrence of ISC are shown with a circle, BSR with a rounded rectangle, DCC and DWC with an ellipse and WCC with a rectangle. (**f**)–(**j**), same as (**a**)–(**e**), but for positive TBO years. (**k**)–(**o**), same as (**a**)–(**e**), but for negative TBO phase. Here, the India map outlines are plotted using MATLAB R2019b programming language.

The long-term (June to September of 1998 to 2013) TRMM observations are well suited to study the monsoon convective characteristics due to their high spatiotemporal coverage^15^. Hence, we attempt to understand the convective characteristics during TBO phases using TRMM dataset. The background meteorological environment (like SST, sea level pressure, 850 hPa wind) associated with different phases of TBO are provided in the supplementary material. The convective storm population varies spatially in positive and negative TBO periods. Figure  1 shows the spatial variations of different convective system’s (ISC, BSR, DCC, WCC and DWC) climatology as well as in two TBO phases. These spatial structures are generated using uneven number of positive (six) and negative (four) TBO years. Thus, the data is normalized using total number of occurrences of all convective systems at each grid point over the study duration. This overview the fractional occurrence of different convective systems during the monsoon season. Among all categories, BSR (Fig. 1b) is the most dominant convective system in the Indian monsoon region. The ISC (Fig. 1a), which can be understood as the individual packets of shallow cumuli, form preferentially along the west coast of India and AS (shown with a circle). The shallow nature of convection over India’s west coast and their contribution to heavy orographic rainfall have been previously explored using ground-based and space-borne radar measurements^7,19,21,39,40^. This remain the subject of interest in the recent decades due to limited understanding on fundamental physical mechanisms.

The most frequent convective system among all, BSR echoes (Fig. 1b) form over the northern and eastern parts of the BoB and orographic regions of the Myanmar (Arakan Yoma) coast (shown with a rounded rectangle) during the ISM. The BSR echoes are also observed in central India (rounded rectangle) and in the southern parts of AS but with relatively fewer occurrences and smaller spatial extent. The DCC (Fig. 1c) and DWC (Fig. 1e) echoes preferentially occur along the foothills of Himalayas (shown with an ellipse) with maxima along the western rim. The DWC echoes are also present along east coast of southern peninsula and BoB, however, their occurrence is small. The occurrence of WCC (Fig. 1d) is observed in the eastern BoB, Myanmar coast (shown with a rectangle) and along the western coast. These results are consistent with Romatschke et al.^11^, where they observed a higher frequency of DCC in the western foothills of Himalayas and BSR in the eastern parts of the BoB.

### Convective systems spatial distribution over Indian subcontinent during positive and negative TBO periods

**Figure 2:**
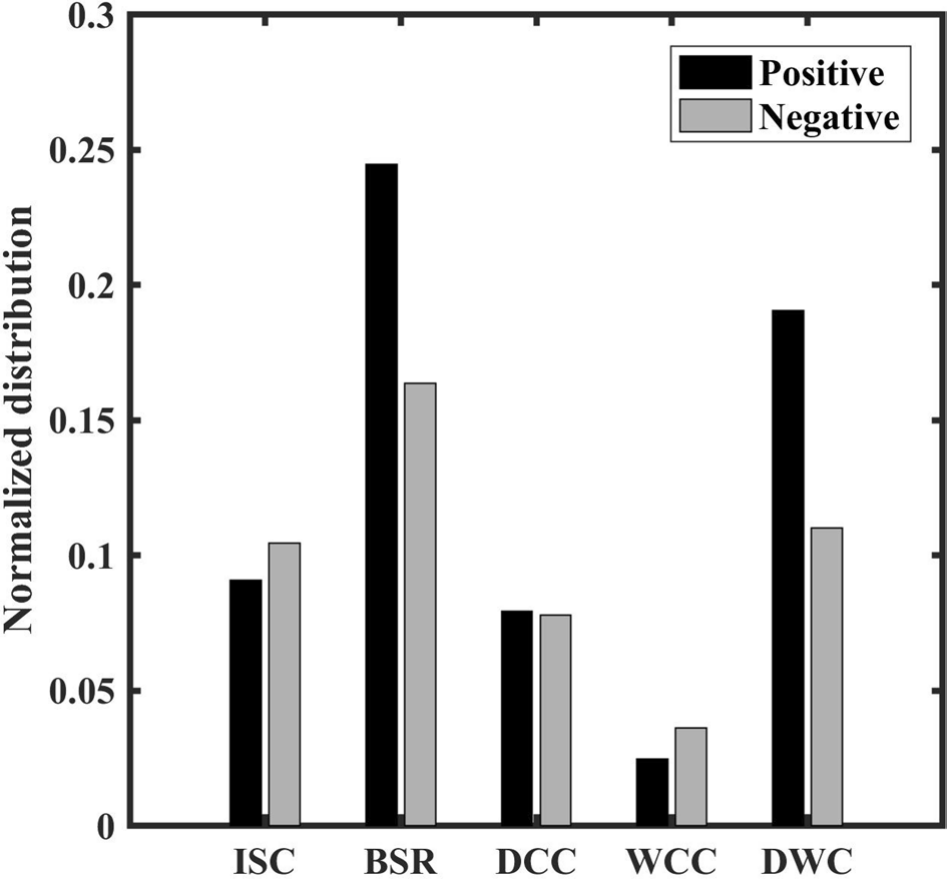
Histogram of normalized occurrence of different convective systems during positive and negative TBO phases. Here, the histograms are calculated for the regions marked in Fig. 1.

The spatial distribution of convective storms during positive (Fig. 1f–j) and negative (Fig. 1k–o) TBO phases shows similar variability in terms of climatology but distinct distribution for different classes of convective systems. Therefore, the convective variability and their modulation by TBO phases will be discussed separately for each type of convective system. Looking into the ISC, the spatial distribution during both the phases shows maxima in AS and along the west coast with a slightly higher frequency during negative TBO. The occurrence frequency of WCC is higher in the BoB and Myanmar coast for the negative TBO phase composite. Similarly, BSR and DWC have spatial inhomogeneity in terms of occurrence distribution, while DCC has a similar spatial pattern during both the phases. Therefore, to better understand the differences in storm frequency during positive and negative TBO phases, the histograms are plotted for the highlighted regions (shown as ellipse, rectangle, and circle in Fig. 1a–e) and shown in Fig. 2. Here, the differences between the occurrence frequency of ISC, WCC and DCC during TBO phases are less but substantial. The maximum difference in the occurrence frequency during TBO phases are observed in the case of BSR and DWC systems. The occurrence of BSR echoes increased over central India and east coast region and BoB during positive TBO years. The foothills of Himalaya’s show a higher frequency of DWC in the positive TBO phase. This implies that the BoB and Indian landmass areas are active during the positive TBO phase, while the AS region is active during the negative TBO phase. The overall findings can be summarized into the observed convective characteristics of different systems as a function of TBO phase. The relatively higher frequent ISC, and WCC during negative TBO compared to positive TBO could be representative of isolated packets of cumuli and widespread shallow MCSs, respectively. More frequent BSR and DWC during positive TBO compared to the negative TBO are representative of deep, wide, and intense MCSs with the possibility of anvil as trailing or outflow.

Now, the question arise here what is the environment during negative TBO phase that could not led the much of shallow populations of convective systems to grow into well-matured deep, wide, and intense MCSs. Therefore, our next aim is to examine the large-scale dynamics and possible causative mechanisms responsible for shallow convection (both isolated cumuli and widespread over AS and BoB) during negative TBO and well-matured MCSs during the positive TBO phase.

### Causative mechanism for the spatial variability of convective systems

**Figure 3:**
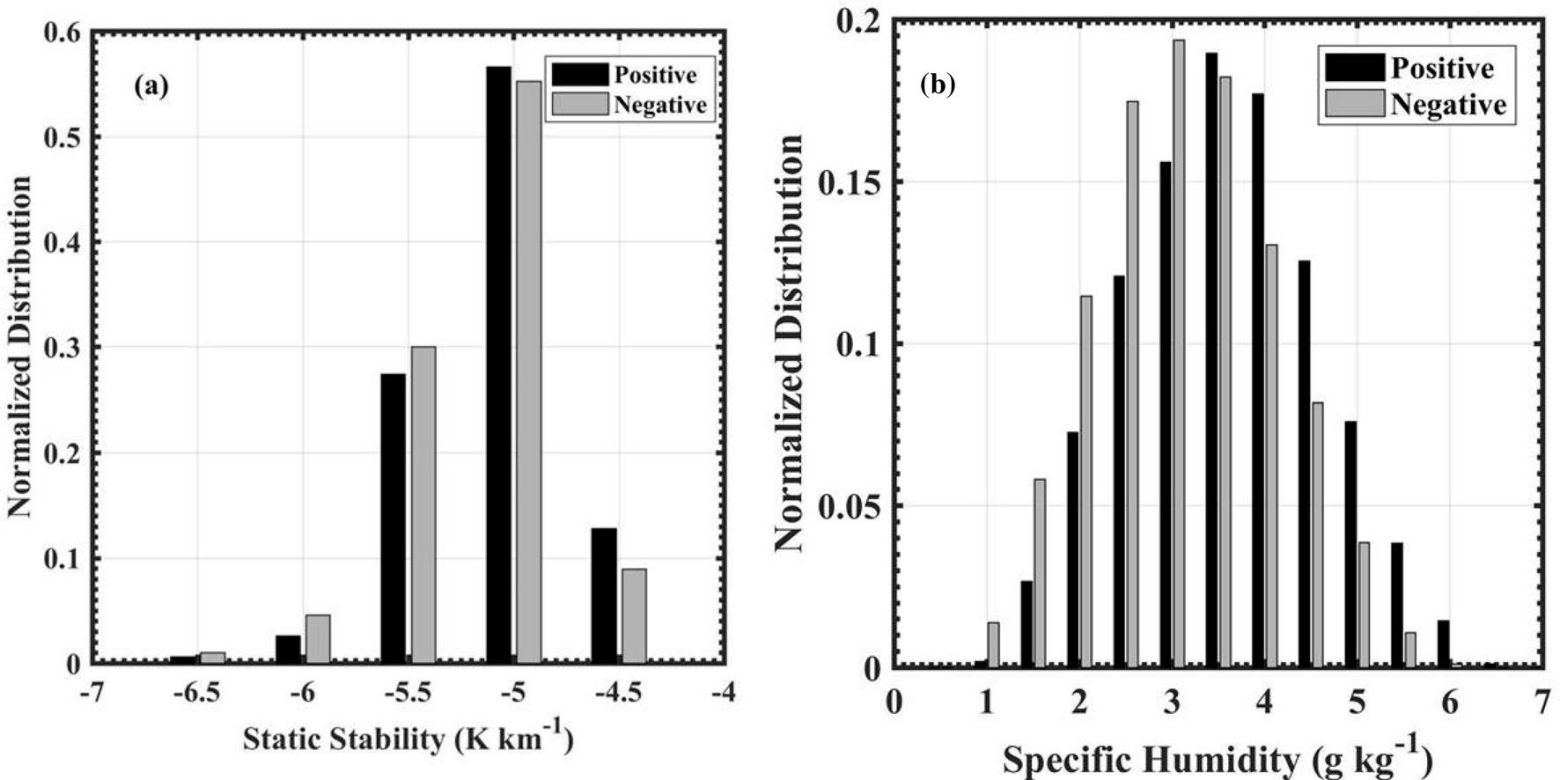
Histograms of (**a**) low-level (1.5–4.5 km) static stability and (**b**) mid-level (4.5–7.5 km) specific humidity for positive and negative TBO phases.

This sub-section examines the atmospheric conditions regulating the convective variability during positive and negative TBO phases. First, we look into the possible large-scale dynamic links to the observed convective variability during the negative phase of TBO. During the negative TBO phase, the AS and Indian west coast exhibit higher frequency of ISC. Based on the existing literature, shallow convection over the Indian monsoon region, particularly the AS and WG, could be due to two plausible mechanisms: statically stable environment suppressing convection depth^19^ and another one is dry mid-level environment limiting the vertical extent of convection^41,42^. Hence, to understand the higher occurrence of ISC during negative TBO phase, the low-level (1.5–4.5 km) static stability and mid-level (4.5–7.5 km) specific humidity are analyzed in the AS and along the west coast of India (60–75$^\circ$E and 10–20$^\circ$N). The histograms of low-level static stability and mid-level specific humidity during positive and negative TBO phases determined over the AS and west coastal region are shown in Fig. 3. The distribution of low-level static stability (Fig. 3a) is negative during both the TBO phases. This indicates the unstable atmosphere in the lower levels during both the TBO phases. However, the mid-level specific humidity (Fig. 3b) distribution is skewed towards lower values during negative TBO phase, indicating a relatively drier atmosphere. Kumar et al.^42^ observed a drier mid-troposphere preceding the formation of congestus clouds during wet seasons over Darwin. Recently, Krishna et al.^21^ observed drier mid-troposphere in coastal and oceanic regions of the Indian west coast, leading to shallow convective storms during mid-night to morning hours. While the static instability support the development of convective systems, the dry mid-troposphere acts as a stronger capping inversion and thereby suppresses the cloud growth resulting in more ISC population over the AS and Indian west coast during negative TBO periods. Nevertheless, the similar negative static stability during both the phases explain that an unstable environment acts as a necessary condition for ISC population while a drier mid-level environment is the sufficient condition for observed convective variability during the negative TBO phase.

The large-scale thermodynamic and dynamic parameters such as low-level potential instability, winds, specific humidity, and vertical integral of divergence of moisture flux are best practiced to understand the young, vigorous, and well matured MCSs (such as DCC, DWC, and BSR). Thus, those variables are analyzed to understand the BSR, DCC, and DWC occurrence mechanisms during different TBO phases. The potential instability in the lower troposphere, defined as the gradient of equivalent potential temperature ($\partial \theta _e$/$\partial Z$) between 1.5 and 4.5 km^19^, is used to understand the regions of active convection. The potential instability describes the atmospheric state where the atmospheric layer becomes statically unstable after lifting. The vertical integral of divergence of moisture flux, quantitative measure of the column integrated moisture influx, is best-suited variable representative of the large-scale dynamics of the atmosphere and is interpreted as the source of enhanced vertical development in the mid-level^43,44^.

**Figure 4:**
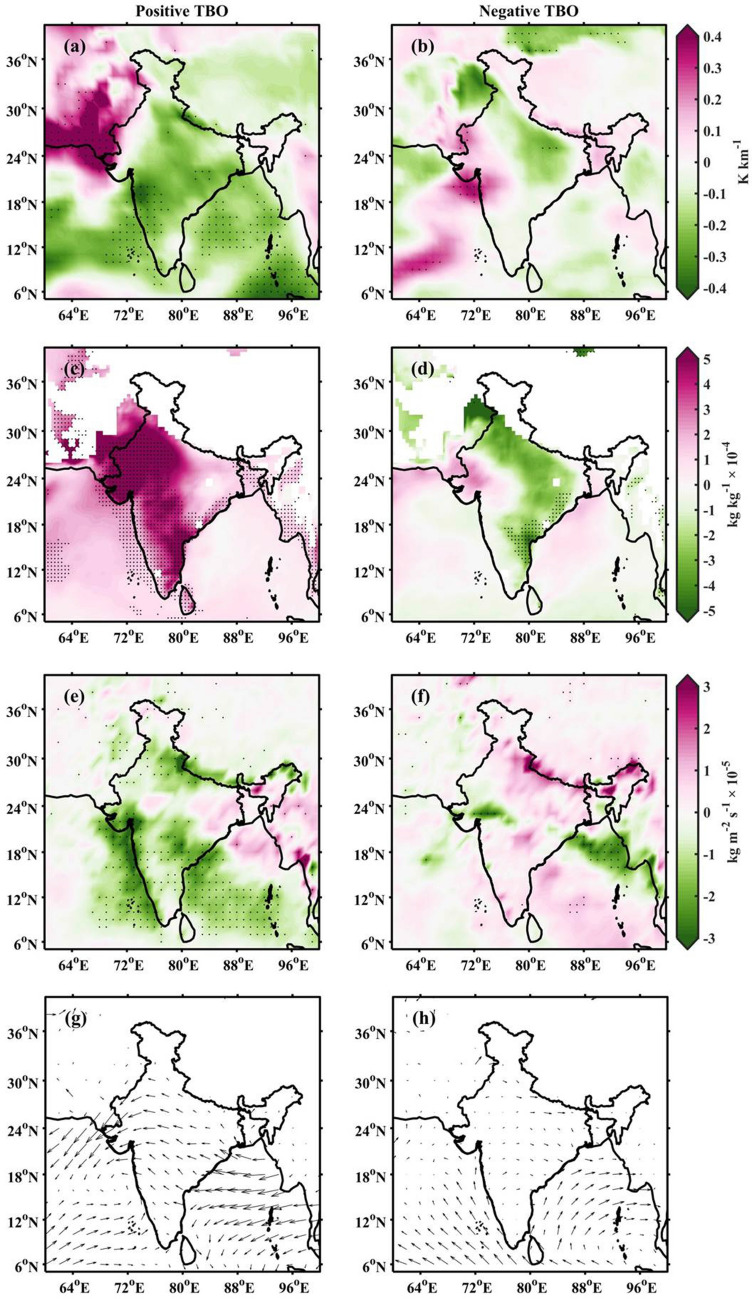
Composite anomalies of low-level potential instability ($\partial \theta _e$/$\partial Z$) during (**a**) positive and (**b**) negative TBO phases. (**c**,**d**), same as (a,b), but for specific humidity at 1000 hPa. (**e**,**f**), same as (a,b), but for vertical integral of divergence of moisture flux. (**g**,**h**), same as (a,b), but for vector wind at 1000 hPa. The black dots in (**a**)–(**f**) represent the data significance at 90$\%$ confidence level using Z-test. Here, the India map outlines are plotted using MATLAB R2019b programming language.

Figure 4a,b present the composite anomalies in potential instability during positive and negative TBO phases. The statistically significant negative anomalies are observed only during the positive TBO phase. This indicates that the atmosphere is convectively unstable during the positive phase and promotes active convection. The specific humidity anomalies at 1000 hPa indicate an increase in specific humidity over the Indian subcontinent during the positive TBO phase (Fig. 4c). The anomalies are significant in the peninsular, central and northwest India. The observed anomalous moist environment could be linked to the observed positive anomalies in SST in the Indian Ocean (Fig. S1). The high SST in the Indian Ocean causes surface evaporation and provides the moisture source for convection in the Indian landmass. The dominance of insignificant anomalies of specific humidity over the Indian region explains little change in moisture availability from mean monsoon conditions during negative TBO phase (Fig. 4d).

TBO is associated with coupled ocean-atmosphere dynamics that involve upper-ocean temperature anomalies. During positive TBO years, the gradient in sea level pressure (SLP, in Fig. S1) induces strong anomalous easterlies in the low-level (1000 hPa) winds over BoB, central and northern India, as shown in Fig. 4g. However, the mean prevailing winds are westerlies in the AS during both TBO phases (Figure not shown). These moisture laden mean westerlies and anomalous easterlies converge and transport moisture to higher levels during positive TBO phase. In comparison, weak westerly anomalies are present over the Indian landmass during negative TBO phase (Fig. 4h), and their interaction with mean westerlies is weak. This is further evidenced by the vertical integral of moisture flux ($\hbox {kg}\,\hbox {m}^{-2}\, \hbox {s}^{-1}$). Figure 4e,f presents the composite anomalies in the vertical integral of divergence of moisture flux. Here, negative values indicate a decrease in divergence or an increase in convergence, and positive values represent vice versa. The anomalous moisture convergence can be observed along the Himalayan foothills, central India and southern parts of BoB during positive TBO years. The low-level convergence between monsoon westerlies and the anomalous easterlies promotes BSR echoes in the BoB, central India and east coast region during positive TBO years. The stratiform echoes in the BoB and adjoining areas could be associated with synoptic-scale low pressure systems developing in the BoB^10^. The observed WCC and BSR over eastern parts of the BoB and Myanmar coast during negative TBO years can be explained in terms of anomalously higher columnar moisture flux (Fig. 4f).

**Figure 5:**
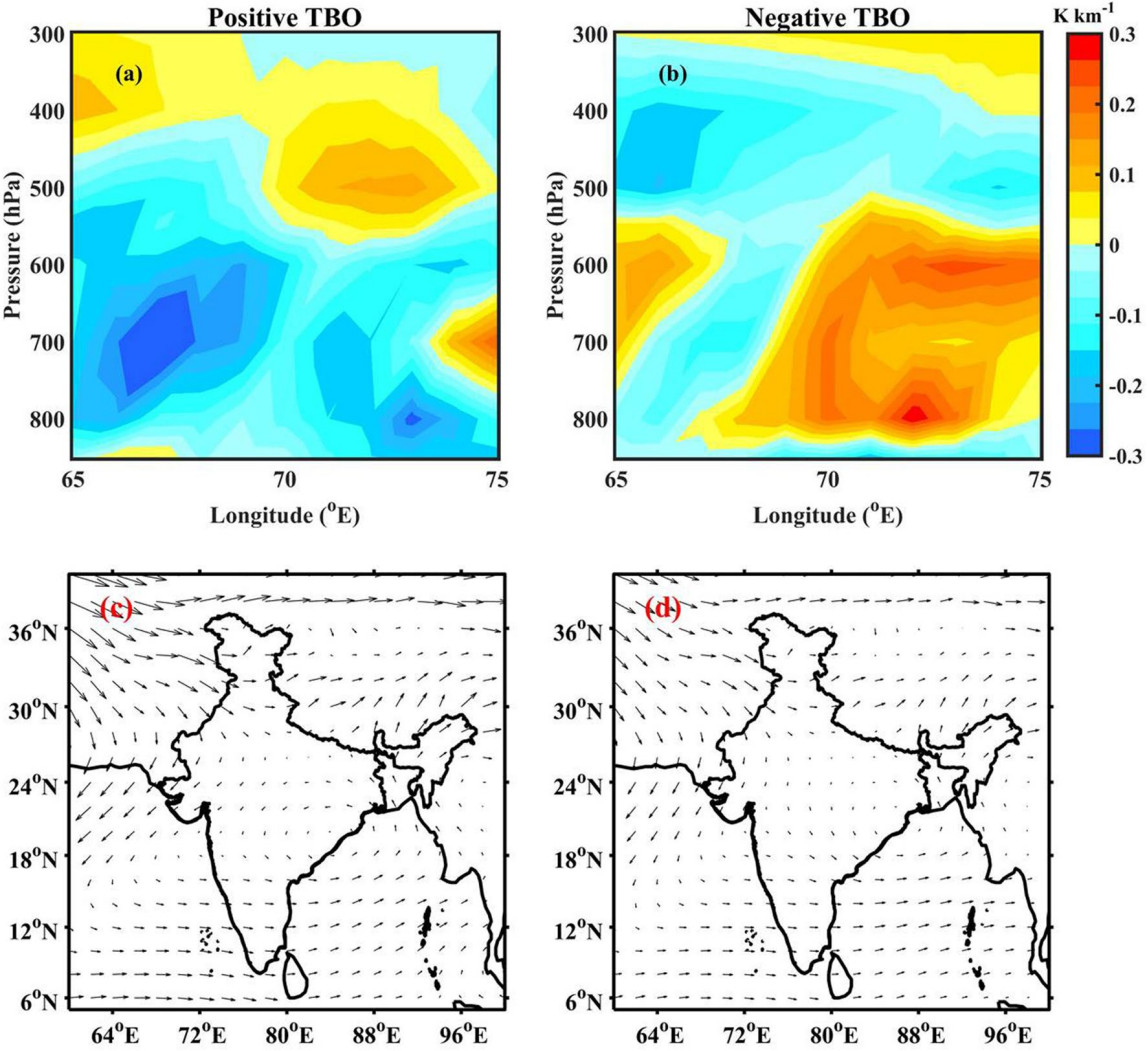
Zonal-vertical cross-section of potential instability over western Himalayan foothills (in the latitude box 28-$35^\circ$N) during (**a**) positive and (**b**) negative TBO years. The composite wind direction (black arrows) at 500 hPa during (**c**) positive and (**d**) negative TBO phases. The India map outlines in (**c**) and (**d**) were created using the MATLAB R2019b programming language.

**Figure 6:**
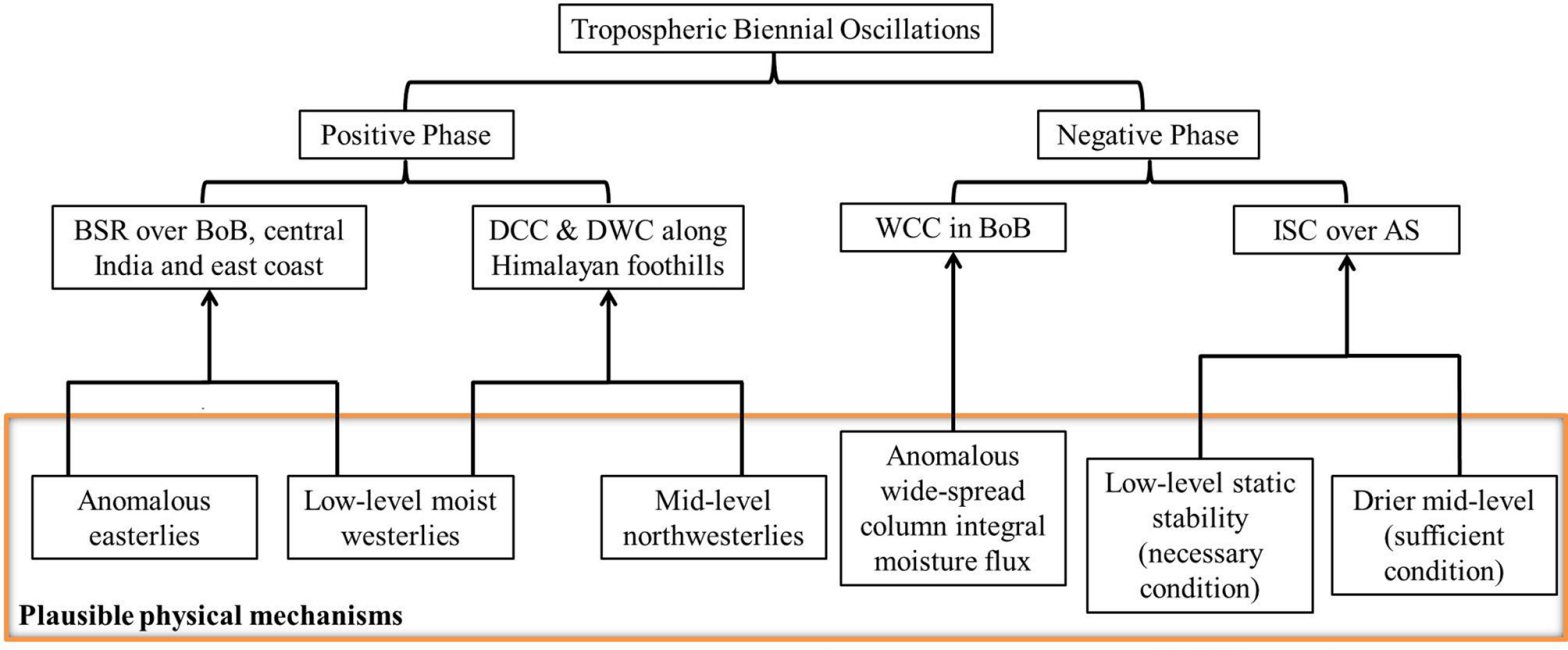
Summary of plausible mechanism for convective storm spatial variations during different TBO phases.

The formation of deep convection requires three ingredients: (i) favourable environment, i.e., a conditionally unstable atmosphere, (ii) sufficient lower- and mid-troposphere moisture and (iii) triggering mechanism^45^. The potential instability is estimated over the western Himalayan foothills (28–$35^\circ$N and 65–$75^\circ$E) and shown as a zonal-vertical cross-section during positive and negative TBO phases in Fig. 5a,b, respectively. The potential instability is negative in the lower levels (upto 500 hPa), indicating unstable atmosphere over the western Himalayan foothills region during positive TBO phase. Further, a stable layer exists in the mid-levels (500–300 hPa). This could be due to the dry north-westerlies from Afghan mountains, which bring dry air at 500 hPa as shown in Fig. 5c. Over the Himalayan region, Houze et al.^10^ found that the dry westerlies play a significant role in the frequent formation of wide convection during monsoon season. The mean monsoon westerlies in the lower levels with abundant moisture from AS transport moisture flux (Fig. 4e) near the Himalayan foothills during positive TBO. Therefore, the moist westerly in the lower levels are topped with the dry north-westerly winds. While westerly in the lower levels continuously feed moisture, north-westerly dry air from Afghan mountains acts as capping inversion^46^. A conditionally unstable environment coupled with the continuous moisture supply in the lower levels capped with dry air above builds energy over a period. As soon as some external triggering mechanism (orography in this case) lifts the air parcel sufficiently to overcome the capping (stable) layer above, in turns deep and intense convection form in the foothills^10,46^. In addressing the convective processes over the Himalayan region, Hunt et al.^47^ observed that the low-level flow brings the moisture to the foothills region and, with a subsequent forced ascent supports the formation of deep convection along the Himalayan foothills. So, the low-level moisture flow intrudes on the Himalayan foothills, the moist and unstable air is orographically lifted to break through the mid-level stable layer and releases its instability and forms DWC during positive TBO years^10,46^. However, during the negative TBO phase, the atmosphere is stable in the lower levels (up to 550 hPa), as indicated by the vertical cross-section of potential instability (Fig. 5b). In addition, the dry north-westerly flow can also be seen in the mid-level during negative TBO phase (Fig. 5d). These conditions suppress the development of convective systems over the western Himalayan region during negative TBO phase. The plausible mechanisms for the observed spatial variability in convective storms during different TBO phases are summarized in Fig. 6.

## Summary

The present study aims to understand the spatial variations in the convective systems during positive and negative phases of TBO in the Indian subcontinent. For this, long-term (June to September of 1998–2013) TRMM-PR data classified into different convective categories by Houze et al.^10^ is used. The different convective systems show distinct spatial variability over the Indian monsoon regime. The salient features of the present study are:

i. A relatively higher ISC occurrence is over AS and the west coast of India during negative TBO phase. The drier mid-troposphere plays a crucial role in inhibiting cloud growth during negative TBO phase, which leads to the formation of ISC over AS and the adjoining west coast region.
ii. The frequency of BSR echoes is higher in the continental regions and parts of BoB during positive TBO years. When the mean monsoon westerlies converge with anomalously unstable (convectively) easterlies from BoB during positive TBO years, it triggers the BSR echoes.
iii. The WCC echoes present along orographic regions, i.e., in the eastern parts of BoB, Myanmar, and the Indian west coast during negative TBO years.
iv. The occurrence of DWC is higher along the foothills of Himalayas during positive TBO years. The moist low-level monsoon flow capped by dry mid-level flow from high latitudes create a stable layer in mid-level. Further, the orographic lifting along the foothills breaks through this mid-level stable layer, releases its instability, and forms DCC and DWC during positive TBO years.

The spatial distribution of convective systems and the plausible large-scale dynamic processes governing different convective systems across the Indian monsoon region are investigated in this work. Apart from TBO, ocean-atmosphere linked phenomena such as ENSO, intra-seasonal oscillations, quasi-biweekly oscillation, and convectively coupled waves (Rossby, Kelvin, etc.) interact with one another, modulating synoptic conditions and resulting in regional variability in convective strength. Although we attempt to examine the modulation of convective variability by TBO modes, it is likely that the other mode of oscillation (low-to-high frequency oscillations) could also be responsible for modulating the convective variability of these systems during monsoon. Therefore, the observed similar convective characteristics during both the phases such as in case of DCC, and/or less variable nature of convection as function of TBO mode (e.g., ISC, WCC) put forward the clue on the relatively stronger role of other frequency modes for monsoon convection variability. Investigating these characteristics in the context of monsoon rainfall is intriguing and understudied and can be considered for future studies.

This study further revealed the global teleconnections that contribute to Indian rainfall variability. Incorporating these characteristics and any potential teleconnections into climate models can improve the simulation of the Indian summer monsoon rainfall under the effect of these phenomena. This is an interesting research topic which needs further investigation. Future study includes the modelling aspects of improving Indian summer monsoon simulations by including Indo-Pacific ocean teleconnections.

## Data and methodology

The TBO signals can be detected in the Indian rainfall during the summer monsoon season (June to September). Konda et al.^38^ analyzed the TBO rainfall using observations and model simulations. They classified the ISM rainfall into positive and negative TBO phases by analyzing the area-averaged rainfall anomalies over 5–$40^\circ$N and 60–$100^\circ$E. If the area-averaged rainfall anomaly for a given year is higher than the previous and following year, it is considered a positive TBO year. Similarly, if the area-averaged rainfall anomaly is smaller than the previous year and the following year, it is a negative TBO year. We found 7 positive TBO years (1988, 1998, 2003, 2005, 2007, 2010, and 2013) and 5 negative TBO years (1997, 2004, 2006, 2009, and 2012) during the study period.

TRMM-PR measurements are analyzed for 16 years from 1998 to 2013 over India and adjoining oceans ($5^\circ$–$40^\circ$N and $60^\circ$–$100^\circ$E) to examine the convective characteristics during positive and negative TBO years. Houze et al.^10^ and Romatschke et al.^11^ classified the convective systems into five categories: isolated shallow convection (ISC), broad stratiform rain (BSR), deep convective core (DCC), wide convective core (WCC), and deep wide convection (DWC) using TRMM data and are archived at http://trmm.atmos.washington.edu. The classification criterion for the TRMM data uses 30 dBZ top heights and horizontal extent of convective storms by utilizing the level 2 PR products, 2A23 (rain types, Awaka et al.^48^), 2A25 (reflectivity and rainfall rate, Iguchi et al.^49^), and level 3 product, 3A25 (gridded rainfall, Meneghini et al.^50^). The classification details can be found in Houze et al.^10^. The data are available at $0.05^\circ$ latitude-longitude and 250 m resolution vertically. In this study, the convective systems are characterized during six positive TBO years (1998, 2003, 2005, 2007, 2010, and 2013) and four negative TBO years (2004, 2006, 2009, and 2012). The convective systems classified by Houze et al.^10^ and Romatschke et al.^11^ are used to understand the spatial variation in the convective characteristics during positive and negative TBO years. The convective systems are segregated into $1^\circ \times 1^\circ$ latitude and longitude bins. The occurrence frequency for each bin is calculated if the total number of rainy pixels in that bin exceeds 500, as discussed in Saikranthi et al.^15^. The occurrence of any convective system, for e.g., BSR, at a given $1^\circ \times 1^\circ$ (latitude $\times$ longitude) bin is calculated by accounting for the number of BSR pixels in that bin and taking the ratio with respect to the total number of rainy pixels observed in that bin obtained from 3D interpolated grid that is multiplied by 100. In the present study, the convectived systems classified using moderate threshold^51^ is utilized.

The TBO years chosen for this study could be influenced by ENSO signals as the frequency of both the oscillations are on interannual scales. Therefore, it is likely that ENSO could also contribute to the variability in the convective storms. To separate the ENSO signal from the TBO, the convective storm occurrence is calculated for TBO years by excluding ENSO years and is shown in the supplementary material (Fig. S3). The convective systems show similar variability but with different amplitudes even after filtering the ENSO years.

Furthermore, to examine the background mechanisms responsible for the observed differences in the convective features during different TBO years, ERA5 data are utilized. ERA5 (Copernicus Climate Change Service, C3S, https://www.ecmwf.int/en/forecasts/datasets/reanalysis-datasets/era5) is the most extensive global atmospheric reanalysis product produced by the European Center for Medium-range Weather Forecasting (ECMWF)^52^. ERA5 data are available in near-real-time from 1 January 1979 to present. ERA5 generates hourly and monthly gridded data over the globe, including a large variety of atmospheric parameters at the surface and vertical that describes the weather. The ERA5 monthly mean temperature, specific humidity, zonal and meridional wind, and vertical integral of divergence of moisture flux, which is available at different pressure levels and 0.25$^\circ$ spatial resolution, is utilized.

To investigate the plausible causative mechanisms for the observed frequencies in convective systems, the dynamical (vertical integral of divergence of moisture flux), thermodynamical (lower- and mid-tropospheric humidity) and stability (static stability and potential instability in the lower-troposphere) parameters are analyzed during positive and negative TBO years. The low-level (between 1.5 and 4.5 km) static stability and potential instability are calculated as

1 $$\begin{aligned} \frac{\partial X}{\partial Z}= \frac{X(Z_{i+1})-X(Z_i)}{ Z_{i+1}-Z_i} \end{aligned}$$

Here, *X* refers to virtual temperature ($\hbox {T}_v$) and equivalent potential temperature ($\theta _e$), $\hbox {Z}_{i+1}$ and $\hbox {Z}_i$ refers to the upper and lower level, respectively.

### Ethics declarations

Not applicable.

### Consent to participate/Consent to publish

All authors agreed for publication.

## Supplementary Information

Supplementary Information.

## Data Availability

The datasets generated and/or analysed during the current study are available in http://trmm.atmos.washington.edu, https://www.ecmwf.int/en/forecasts/datasets/reanalysis-datasets/era5, and https://psl.noaa.gov/data/gridded/data.ncep.reanalysis.html.
