# Supplementary material for: Dynamical links of convective storms associated with tropospheric biennial oscillation in the Indian monsoon regime
Source: Sci Rep. 2022 Jul 14;12:12050. doi: 10.1038/s41598-022-15772-9 (PMC9283390; doi:10.1038/s41598-022-15772-9)
Supplement: Supplementary file 1 — Supplementary Information. [file 41598_2022_15772_MOESM1_ESM.pdf]

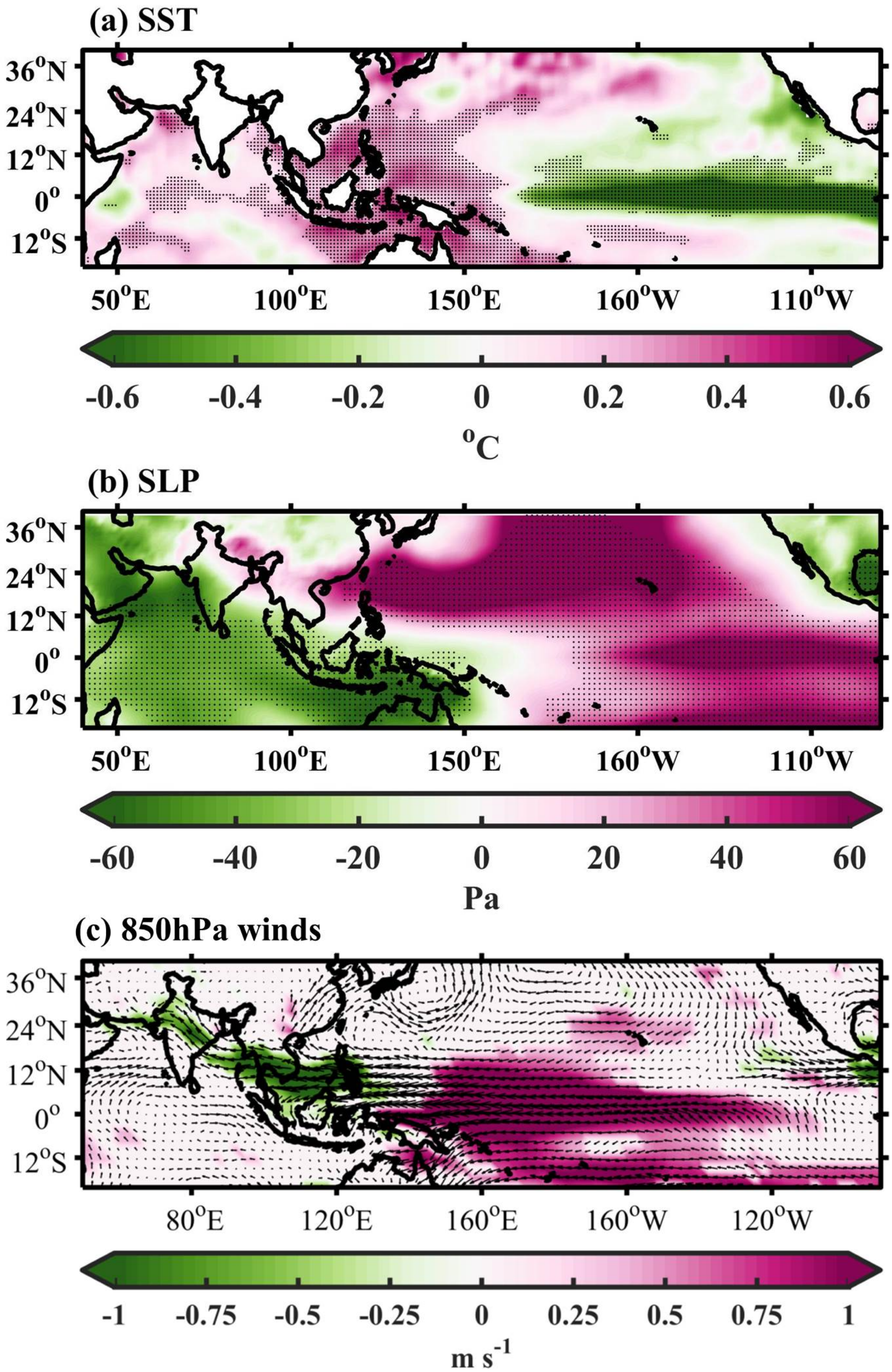

Figure S1: The anomalies of (a) sea surface temperature (SST; °C), (b) sea level pressure (SLP; Pa), and (c) wind speed at 850 hPa (m s^{-1}) during positive TBO periods. The arrows in (c) represent the wind vector, and shading is shown for above 90% significance. The black dots in (a)-(b) represent significance at 90% confidence level using Z-test. The SST data are from the NCEP-NCAR reanalysis `https://psl.noaa.gov/data/gridded/data.ncep.reanalysis.html`, from 1986 to 2015 and SLP and winds are from ERA5 `https://www.ecmwf.int/en/forecasts/datasets/reanalysis-datasets/era5`.

.

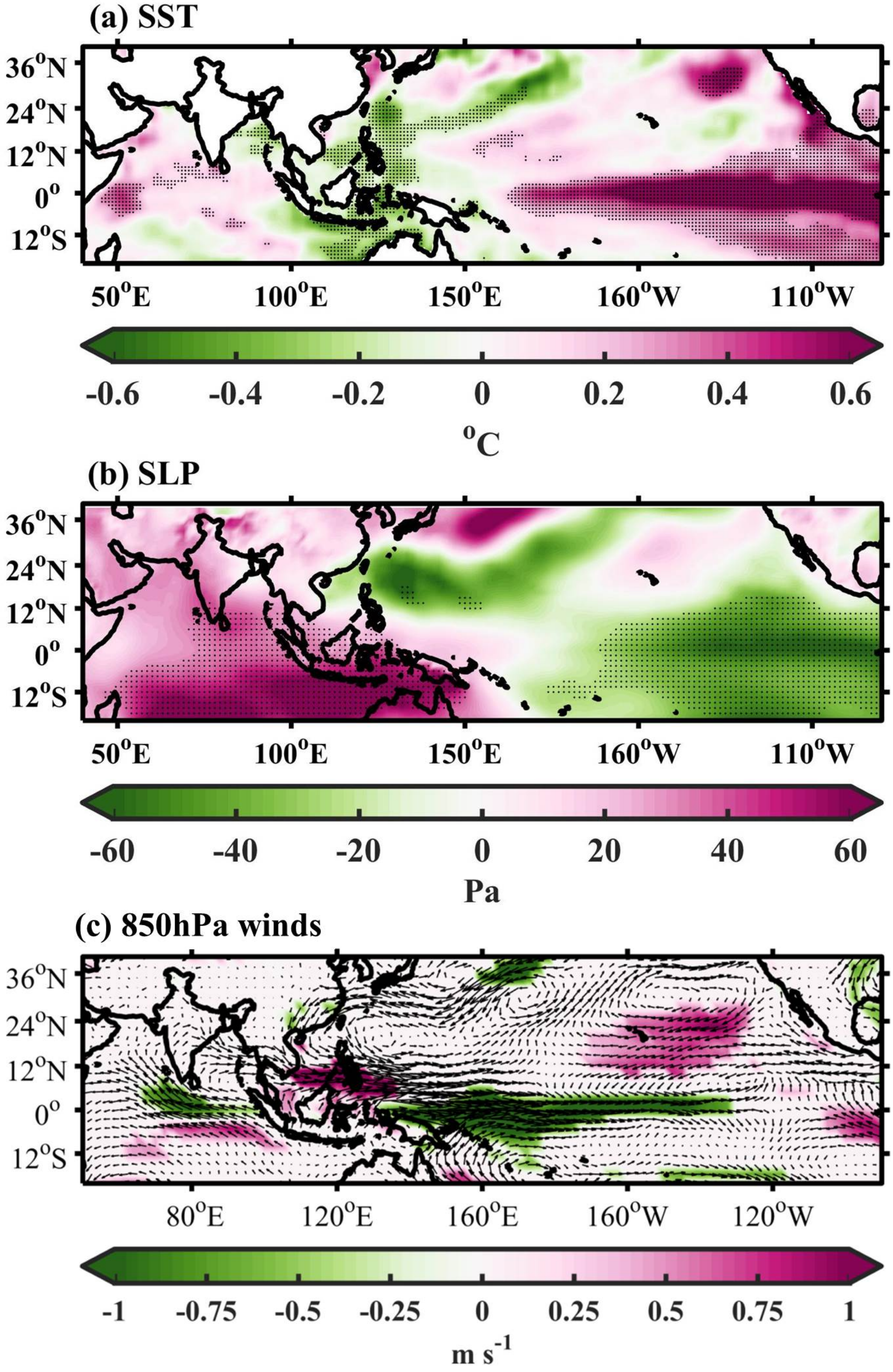

Figure S2: Same as Fig. S1, but for negative TBO years.

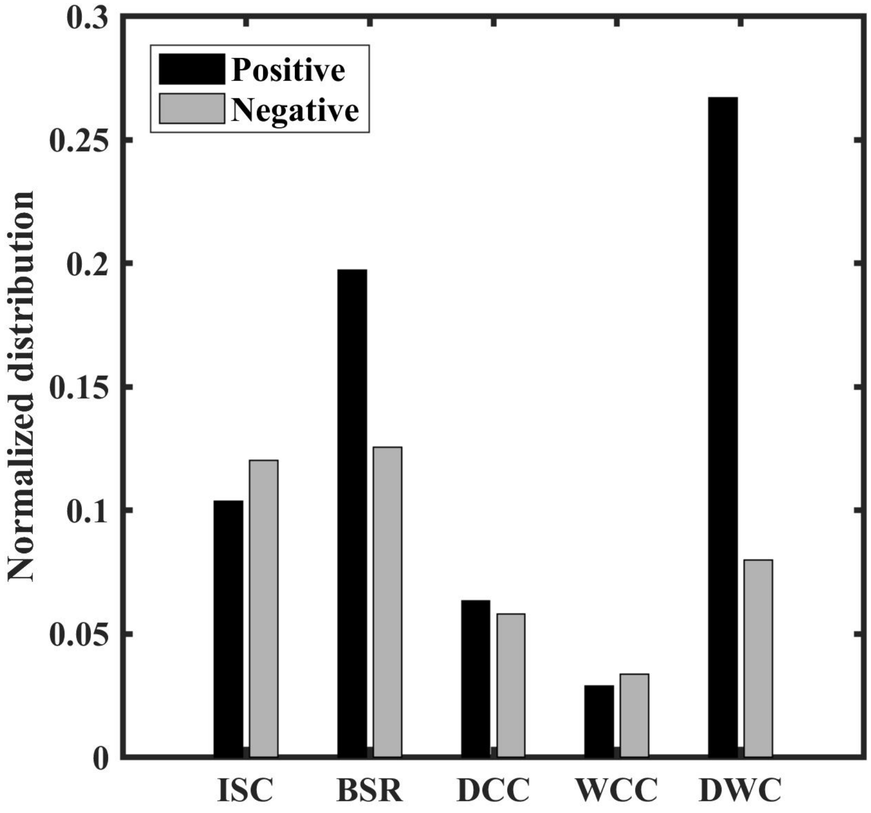

Figure S3: Histogram of normalized occurrence of different convective systems during positive and negative TBO phases by eliminating the ENSO years.
